# ASSVd infection inhibits the vegetative growth of apple trees by affecting leaf metabolism

**DOI:** 10.3389/fpls.2023.1137630

**Published:** 2023-02-24

**Authors:** Guofang Li, Jinghong Li, He Zhang, Jiuyang Li, Linguang Jia, Shiwei Zhou, Yanan Wang, Jianshe Sun, Ming Tan, Jianzhu Shao

**Affiliations:** ^1^ College of Horticulture, Hebei Agricultural University, Baoding, China; ^2^ Changli Institute of Pomology, Hebei Academy of Agricultural and Forestry Science, Changli, China; ^3^ College of Plant Protection, Hebei Agricultural University, Baoding, China

**Keywords:** apple, ASSVd, tree phenotype, photosynthesis, nutrient element, metabolome

## Abstract

Apple scar skin viroid (ASSVd) can infect apple trees and cause scar skin symptoms. However, the associated physiological mechanisms are unclear in young saplings. In this study, ASSVd-infected and control ‘Odysso’ and ‘Tonami’ apple saplings were examined to clarify the effects of ASSVd on apple tree growth and physiological characteristics as well as the leaf metabolome. The results indicated that leaf ASSVd contents increased significantly after grafting and remained high in the second year. Leaf size, tree height, stem diameter, branch length, and leaf photosynthetic efficiency decreased significantly in viroid-infected saplings. In response to the ASSVd infection, the chlorophyll a and b contents decreased significantly in ‘Odysso’, but were unchanged in ‘Tonami’. Moreover, the N, P, K, Fe, Mn, and Ca contents decreased significantly in the leaves of viroid-infected ‘Odysso’ or ‘Tonami’. Similarly, the CAT and POD contents decreased significantly in the viroid-infected saplings, but the SOD content increased in the viroid-infected ‘Tonami’ saplings. A total of 15 and 40 differentially abundant metabolites were respectively identified in the metabolome analyses of ‘Odysso’ and ‘Tonami’ leaves. Specifically, in the viroid-infected ‘Odysso’ and ‘Tonami’ samples, the L-2-aminobutyric acid, 6″-O-malonyldaidzin, and D-xylose contents increased, while the coumarin content decreased. These metabolites are related to the biosynthesis of isoflavonoids and phenylpropanoids as well as the metabolism of carbohydrates and amino acids. These results imply that ASSVd affects apple sapling growth by affecting physiological characteristics and metabolism of apple leaves. The study data may be useful for future investigations on the physiological mechanisms underlying apple tree responses to ASSVd.

## Introduction

Apple scar skin viroid (ASSVd) can decrease the marketability of apple fruits, with detrimental effects on the apple industry. Specifically, ASSVd infections can decrease the size of apple fruits and lead to scarring and staining of the fruit peel (Koganezawa, 1985; Kim et al., 2010; Lee et al., 2017; Li et al., 2021). The incidence of ASSVd infections in the main apple-producing regions in Northern China, reportedly ranges from 4.8% to 48.6% (Zha et al., 2022). For the transmission of ASSVd, the most common mode is contact transmission (e.g., mechanical and agricultural operations) (Verhoeven et al., 2010). In terms of apple, the spread of scions, rootstocks, and saplings during asexual propagation is the main route for ASSVd transmission. It has been detected in various apple tissues (e.g., leaves, stems, skins, stocks, fruits, and roots) (Desvignes et al., 1999; Walia et al., 2014; Wu et al., 2015; Kim et al., 2019). However, symptoms of ASSVd infection are not appeared on the apple leaves and stems (Kim et al., 2010; Wu et al., 2015; Di Serio et al., 2018).

Replication of the ASSVd, which is a noncoding RNA molecule consisting of ~330 nucleotides, rely on host functional enzymes through an RNA-to-RNA rolling circle mechanism (Flores et al., 2009; Li et al., 2021). In order to establish systemic infection, these RNAs can traffic from an initially infected cell into neighboring cells and ultimately throughout a whole plant *via* plasmodesmata and phloem (Takeda and Ding, 2009). The method used for detecting ASSVd mainly involves the identification of botanical symptoms and the application of molecular techniques (Heo et al., 2019; Li et al., 2020). Because of the latent incubation period after ASSVd infection, and some infected plants lack obvious symptoms (Desvignes et al., 1999; Wang et al., 2000; Jiang et al., 2017), which may be difficult to identify ASSVd infection with botanical symptoms. The previously established quantitative real-time PCR (qRT-PCR) assay for detecting ASSVd is more sensitive and rapid than the alternative methods (e.g., conventional RT-PCR, spot hybridization, and botanical identification) (Wu et al., 2015; Xi et al., 2020; Heo and Chung, 2021). Plant metabolomics research mainly involves qualitative and quantitative analyses of metabolic processes under certain stress conditions to elucidate specific biological activities (Han et al., 2019). For example, the examination of the phloem of Verticillium wilt-infected mulberry plants revealed changes in various sugar, amino acid, and organic acid metabolites (Gai et al., 2014). The changes in the phloem sap composition were greater than the changes detected in leaves. The analysis of the poplar metabolome indicated several metabolites, such as p-Octylphenol, plant alcohol, catechol, and eugenol, are related to rust resistance (Zhou et al., 2013). Hence, exploring the apple metabolome may lead to the identification of metabolites responsive to an ASSVd infection.

In this study, ‘Odysso’ and ‘Tonami’ apple saplings were selected as materials for an investigation of the ASSVd content in growing saplings and its influence on young sapling physiological characteristics. Furthermore, leaf metabolomics experiments were conducted to systematically evaluate the effects of ASSVd on young sapling growth and physiological characteristics. The generated data were used to elucidate the mechanism by which ASSVd damages apple saplings. The findings of this study may provide the theoretical basis for future studies conducted to clarify the mechanism underlying ASSVd infections, while also highlighting the necessity of virus-free cultivation.

## Materials and methods

### Plant materials

This study was carried out at the fruit demonstration station of Hebei Agricultural University, Shunping county, Hebei province (38.97N, 114.92E), China. The newly grafted (NG) axillary buds and 1-year-old (OY) saplings examined in this study were from ‘Odysso’ (red-fleshed apple fruits with high anthocyanin contents bred by Markus Kobelt in Switzerland) and ‘Tonami’ (*Malus domestica* ‘Tonami’) apple cultivars. The virus-free *M. robusta* rootstocks used for grafting were rapidly propagated in a tissue culture system. Viroid-infected ‘Odysso’ was obtained by grafting onto ‘Tonami’ apple trees infected with ASSVd. Axillary buds on the annual branches of all scions were collected at the Hebei Agricultural University nursery in early March. The ‘Odysso’ and ‘Tonami’ saplings infected with ASSVd (i.e., treatments) were compared with the virus-free control ‘Odysso’ and ‘Tonami’ saplings. The grafted saplings were transferred to 45 cm plastic pots. Ten saplings were used per treatment. All saplings were grown under consistent conditions.

### Quantitative real-time PCR analysis of ASSVd and production of a standard curve

Total RNA was extracted from sapling leaves using the polysaccharide polyphenol plant total RNA extraction kit (Tiangen, Beijing). The integrity, purity, and concentration of the isolated RNA were determined on the basis of the A260/A280 and A260/A230 ratios using the NanoDrop 2000 spectrophotometer (Thermo Scientific, USA). The RNA served as the template for synthesizing cDNA using the TransScript One-Step gDNA Removal and cDNA Synthesis SuperMix kit (TransGen Biotech, Beijing). The qRT-PCR assays were completed using the LightCycler^®^ 96 PCR system (Roche, Switzerland) as previously described (Wu et al., 2015). We performed qRT-PCR with a SYBR Premix Ex Taq II kit (Takara, Beijing) in a 25-μl reaction system. The cycling protocol consisted of 95 °C for 30 s, followed by 39 cycles of 95 °C for 15 s, 60 °C for 20 s and 72 °C for 20 s, followed by 39 cycles to construct a melting curve. The ASSVd sequence-specific primers were developed in our previous studies (**Table S1**, Chen et al., 2012; Wu et al., 2015).

To obtain cRNA, a 106 bp ASSVd fragment was recovered and inserted into the pWASY-T3 vector (TransGen Biotech, Beijing). The recombinant plasmid was transferred into DH5α cells (TransGen Biotech, Beijing), which were screened on LB solid medium containing IPTG and X-gal. The white colonies were subsequently transferred to LB medium supplemented with Ampicillin to obtain single colonies, which were analyzed by PCR. Plasmids were extracted from the colonies with the correct sequence. The extracted plasmid was digested, after which the T7 *in vitro* Transcription Kit was used to generate cRNA.

The standard cRNA solution (1.24 × 10^12^ copies μl^−1^) was serially diluted 10-fold (1.24 × 10^5^–1.24 × 10^11^ copies μl^−1^) for the qRT-PCR conducted to obtain the corresponding linear equation. The data for the biological replicates and replicate tests were used to construct a standard curve, with the cRNA concentration (logarithm value) on one axis and the cycle threshold (Ct) value on the other axis. To assess the reliability of the standard curve, the standard deviation (SD) and variable coefficient (VC) were calculated following the statistical analysis of the Ct values.

### Measurement of tree growth

The tree height was measured from the grafting site using steel tape, whereas the stem diameter was measured at 5 cm above the grafting site using vernier calliper. A portable leaf area meter CI-203 (CID BioScience, USA) and ruler were used to measure leaf parameters. These measurements were performed from April to November for 2 years.

After the leaves fell, the branch compositions were analyzed and the lengths of the short (≤5 cm), medium (5-15 cm), long (15-30 cm), and super-long shoots (>30 cm) were recorded.

### Measurement of photosynthetic parameters and chlorophyll contents in leaves

The net photosynthetic rate (Pn), stomatal conductivity (Gs), transpiration rate (Tr), and intercellular CO_2_ concentration (Ci) of the OY saplings were measured using the LI-6400 Portable Photosynthesis System (Li-Cor, Lincoln NE, USA) on August 10. Five healthy and mature leaves were selected for each treatment. The diurnal changes were measured once every 2 h from 6:00 to 18:00. According to a modified version of an established method (Lichtenhaler and Wellburn, 1983), apple leaves were placed in 10 ml tubes. After adding 5 ml 80% acetone, the tubes were incubated at 4 °C in a black box. The samples were mixed several times every 4 h. As the color intensity of the solutions decreased, the chlorophyll content per unit area was determined by measuring the absorbance at 663 and 645 nm.

### Determination of nutrient elements and antioxidant activities in leaves

For each treatment, 30 healthy and mature leaves were selected, washed, and dried. Following a 30-min incubation at 105 °C, the leaves were dried at 75 °C, crushed, and screened. The ground leaves were collected in a self-sealing bag and stored in a dryer. Leaf samples were boiled in H_2_SO_4_–HClO_4_ and HNO_3_–HClO_4_ solutions and then the nutrient element contents were determined using AutoAnalyzer 3 with XY-2 Sampler (SEAL Analytical, UK) and an inductively coupled plasma emission spectrometer (Prodigy Spec, Teledyne, USA).

On August 10, 10 randomly selected leaves (per treatment) were obtained from OY saplings to measure the catalase (CAT), peroxidase (POD), and superoxide dismutase (SOD) contents as previously described (Wang et al., 2018). CAT was determined by monitoring the decomposition of H_2_O_2_. SOD was assayed by monitoring the inhibition of the photochemical reduction of nitro blue tetrazolium. POD was determined by monitoring the oxidation reaction of guaiacol.

### Extraction, sampling, and analysis of leaf metabolites

Six leaves were collected from ‘Odysso’ and ‘Tonami’ OY saplings, frozen in liquid nitrogen, and stored at −80 °C prior to the leaf metabolomics analysis performed by Novogene Bioinformatics Technology Co. Ltd. The analysis was completed using six biological replicates. Briefly, leaf (100 mg) extracts were prepared using a mixture (methanol: acetonitrile: nitrile water=2:2:1, v/v), after which the 100 µl mixture (acetonitrile: water =1:1, v/v) was added to redissolve the extract. The supernatant was retained for the analysis. Samples were separated using the Agilent 1290 ultra-high performance liquid chromatography system (Agilent, USA) and then analyzed using the TripleTOF 5600 mass spectrometer (AB SCIEX, USA). Accurate quality numbers and secondary spectrograms were used to identify and annotate metabolites according to the Novogene source database.

To evaluate the similarity among biological replicates and the differences between samples, a correlation heatmap was constructed and a principal component analysis (PCA) and a partial least squares discriminant analysis (PLS-DA) were performed on the basis of the metabolite profiles (Lu et al., 2017). A variable importance in projection (VIP) value >1 and *P* < 0.05 were set as the criteria for identifying significant differentially abundant metabolites between samples (Lu et al., 2017; Han et al., 2021). Figures for the heatmaps, volcano maps, Venn diagram, and KEGG pathways were obtained using the Majorbio Cloud Platform (www.majorbio.com).

### Statistical analysis

Significant differences were detected using one-way ANOVA (*P* < 0.05), which was performed using the IBM SPSS Statistics 20. Values in figures represent the mean ± SD. The figures presenting physiological and biochemical data were produced using Microsoft Office Home and Student 2019, whereas the figures presenting the metabolomics data were generated using the Majorbio Cloud Platform.

## Results

### Detection and quantification of ASSVd in ‘Odysso’ and ‘Tonami’ leaves

**Figure 1:**
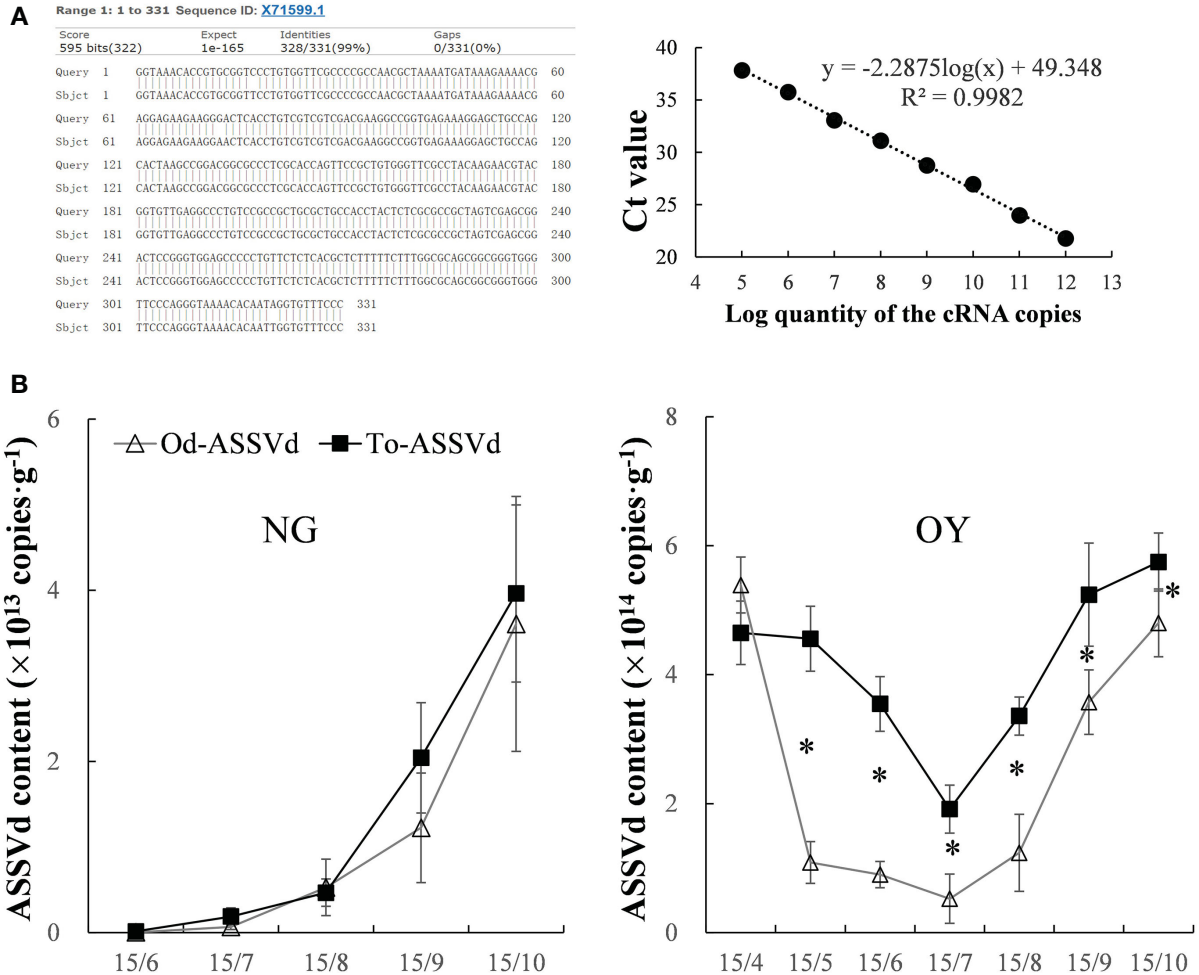
Detection and quantification of ASSVd in leaves. **(A)** Alignment of the most similar sequences revealed by the NCBI BLAST search and standard curve for the qRT-PCR analysis. **(B)** Dynamic changes in the ASSVd content of the NG and OY leaves. Data are presented as the mean ± SD (n=3). The asterisk indicates a significant difference as determined by Student’s *t*-test (*P* < 0.05).

The presence of ASSVd in the leaves of ‘Odysso’ and ‘Tonami’ apple saplings was determined on the basis of gene cloning and a qRT-PCR analysis. The results indicated ASSVd was undetectable in the control ‘Odysso’ and ‘Tonami’ saplings, but it was present in the leaves of infected ‘Odysso’ and ‘Tonami’ saplings (**Figure S1A**). The cloned sequences were analyzed using NCBI BLAST (https://blast.ncbi.nlm.nih.gov/Blast.cgi), which revealed substantial similarities (97.28%–99.09%) to 39 aligned sequences (the most similar sequence was X71599.1; **Figure 1A**, **Figure S1A**). For the quantitative analysis of the viroid levels, the correlation coefficient (R^2^) was 0.998, the amplification efficiency was 90%, and the maximum coefficient of variation among the biological replicates and the replicated tests was 1.35%–1.81% (**Figure 1A**, **Table S2**), which reflected the reliability of the standard curve.

In viroid-infected ‘Odysso’ and ‘Tonami’ NG saplings, the initial ASSVd content was low, but it increased significantly starting on July 15 (approximately the start of the autumn growing season) for both ‘Tonami’ and ‘Odysso’ (**Figure 1B**). In the viroid-infected ‘Odysso’ and ‘Tonami’ OY saplings, the ASSVd content decreased from April 15 to July 15 (after sprouting) and increased from July 15 to October 15 (autumn growing season) (**Figure 1B**). Among the OY saplings, the viroid level was higher in ‘Tonami’ than in ‘Odysso’, except on April 15 (**Figure 1B**).

### Effects of ASSVd on apple sapling leaf size

**Figure 2:**
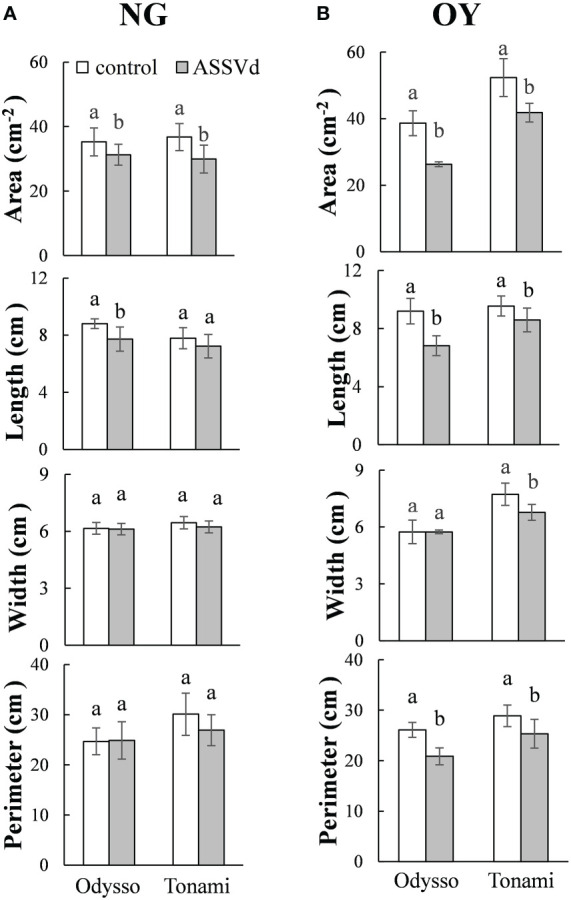
Effects of ASSVd on the size of ‘Odysso’ and ‘Tonami’ leaves. Leaf area, length, width, and perimeter in NG **(A)** and OY **(B)** saplings. Data are presented as the mean ± SD (n=5). Lowercase letters indicate significant differences as determined by Student’s *t*-test (*P* < 0.05).

To quantify the effects of ASSVd on leaf size, leaf area, length, width and perimeter were analyzed in the leaves of NG and OY saplings. Compared with the control, the leaf area and leaf length of the viroid-infected ‘Odysso’ saplings decreased significantly by 11.30% and 12.27% (NG) and 31.95% and 10.63% (OY), respectively (**Figure 2**). The leaf width did not differ between NG and OY. There was also no difference in the NG leaf circumference between the viroid-infected and control ‘Odysso’ saplings. In contrast, the OY leaf circumference of the viroid-infected ‘Odysso’ saplings decreased significantly by 19.99% (**Figure 2**). Compared with control NG saplings, the leaf area and leaf circumference of the viroid-infected ‘Tonami’ saplings decreased significantly by 18.57% and 10.63%, respectively, but there were no significant differences in the leaf length and leaf width (**Figure 2A**). In terms of the OY samples, the leaf area, length, width, and circumference of the viroid-infected ‘Tonami’ saplings decreased significantly by 20.15%, 9.95%, 12.42%, and 12.26%, respectively (compared with the control leaves) (**Figure 2B**). These results indicated that ASSVd can significantly affect the growth of ‘Odysso’ and ‘Tonami’ leaves.

### Effects of ASSVd on the stem height and diameter, and shoot length of grafted scions

**Figure 3:**
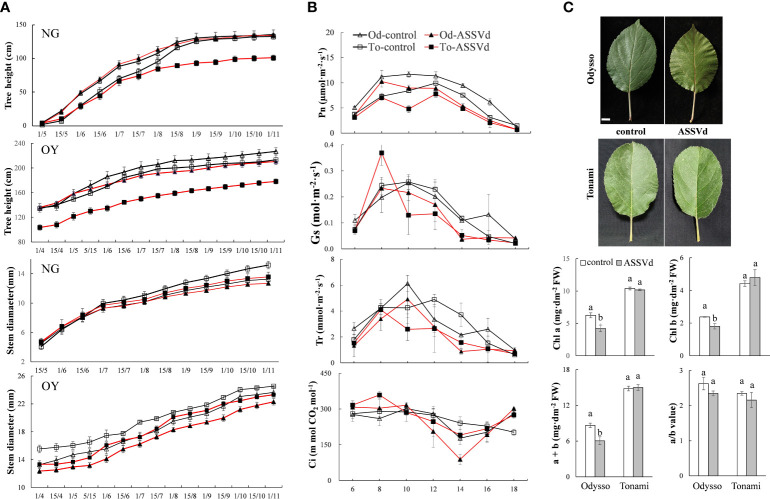
Effects of ASSVd on tree height, stem diameter, photosynthetic activity, and chlorophyll content. **(A)** Tree height and stem diameter curves for the NG and OY saplings of ‘Odysso’ and ‘Tonami’. **(B)** Diurnal variation in the net photosynthetic rate (Pn), stomatal conductance (Gs), transpiration rate (Tr), and intercellular CO_2_ concentration (Ci) in OY leaves. **(C)** Leaf phenotypes and chlorophyll contents in OY leaves. Data are presented as the mean ± SD (n=5). Scale bar = 1.0 cm. Lowercase letters indicate significant differences as determined by Student’s t-test (P < 0.05).

To clarify the effects of ASSVd on shoot growth, stem height and diameter, and shoot length were analyzed in NG and OY saplings. The ‘Odysso’ and ‘Tonami’ NG saplings grew to a similar height. Both cultivars grew rapidly from May to September, but the growth rate decreased after September (**Figure 3A**). In November, there were no significant differences between the control and viroid-infected ‘Odysso’ NG saplings (**Figure 3A**). Before August 1, the differences in the sapling height were insignificant between the control and viroid-infected ‘Tonami’ samples (**Figure 3A**). However, the growth rate and height were significantly greater for the control saplings than for the viroid-infected ‘Tonami’ saplings after August 15 (**Figure 3A**). The increase in ‘Odysso’ and ‘Tonami’ plant height decreased significantly on September 1 and there was essentially no increase by November (**Figure 3A**). Thus, the viroid-infected ‘Tonami’ saplings were significantly shorter than the other three saplings. The comparison between OY and NG indicated the changes in sapling height were generally the same for ‘Odysso’ and ‘Tonami’ (**Figure 3A**). For NG and OY, the stem diameters were similar between ‘Odysso’ and ‘Tonami’ and continued to increase throughout the growing season (**Figure 3A**). The OY sapling stem diameter differed only between the control and viroid-infected ‘Odysso’ samples (**Figure 3A**). However, the NG sapling stem diameter was greater for the control ‘Tonami’ samples than for the viroid-infected ‘Tonami’ samples after August (**Figure 3A**). For the NG and OY saplings, the total branch length (i.e., short, medium, long, and super-long branches and scion height) of the viroid-infected ‘Odysso’ and ‘Tonami’ scions decreased significantly (compared with the control saplings) (**Table S3**).

### Effects of ASSVd on photosynthetic efficiency and the chlorophyll content

The Pn value differed between the viroid-infected and control saplings (**Figure 3B**). In the controls, Pn was highest in ‘Odysso’ and ‘Tonami’ leaves at 8:00–12:00 and 12:00, respectively. For the viroid-infected ‘Tonami’ samples, Pn was lower at 10:00 than at 8:00 or 12:00. In the viroid-infected ‘Odysso’ samples, Pn peaked at 8:00. Generally, Pn was higher in the controls than in the viroid-infected saplings, although Pn was high for both the viroid-infected and control ‘Odysso’ saplings. The Gs value indicates the degree of stomatal opening, which influences the rate of CO_2_ and water vapor exchange. The daily variation curve indicated Gs initially increased and then decreased (**Figure 3B**). The Gs was highest at 8:00 for the viroid-infected ‘Tonami’ saplings, whereas it was highest at 10:00 for the other three materials. In addition, Gs was lower for the viroid-infected ‘Odysso’ and ‘Tonami’ samples than for the corresponding controls at 10:00–16:00. Leaf transpiration is critical for the upward transport of water through plants. The changes in Tr were similar to the Gs trends (**Figure 3B**). The Tr value was highest for the viroid-infected ‘Tonami’ saplings at 8:00, but it peaked for the ‘Odysso’ saplings (control and viroid-infected) and the control ‘Tonami’ saplings at 10:00 and 12:00, respectively. Additionally, for both ‘Odysso’ and ‘Tonami’, Tr was lower for the viroid-infected samples than for the controls at 12:00–16:00. The Ci values fluctuated over the analyzed time-period (**Figure 3B**). The Ci value for the viroid-infected ‘Tonami’ samples was highest at 8:00, whereas it peaked at 10:00 and then decreased significantly to its lowest point at 14:00 for the viroid-infected ‘Odysso’ samples. Chlorophyll a and b are important photosynthetic pigments. Compared with the controls, the chlorophyll a and b contents were significantly higher in the viroid-infected ‘Odysso’ samples, but the chlorophyll a/b ratio was unchanged (**Figure 3C**). In ‘Tonami’, the ASSVd infection did not significantly affect the chlorophyll content and chlorophyll a/b ratio.

The above results showed that the effects of ASSVd on leaf photosynthetic activities and chlorophyll contents differed between ‘Odysso’ and ‘Tonami’. More specifically, the ASSVd infection affected the chlorophyll content in ‘Odysso’, whereas it decreased the photosynthetic efficiency in ‘Tonami’.

### Effect of ASSVd on nutrient elements and antioxidant activities in leaves

**Figure 4:**
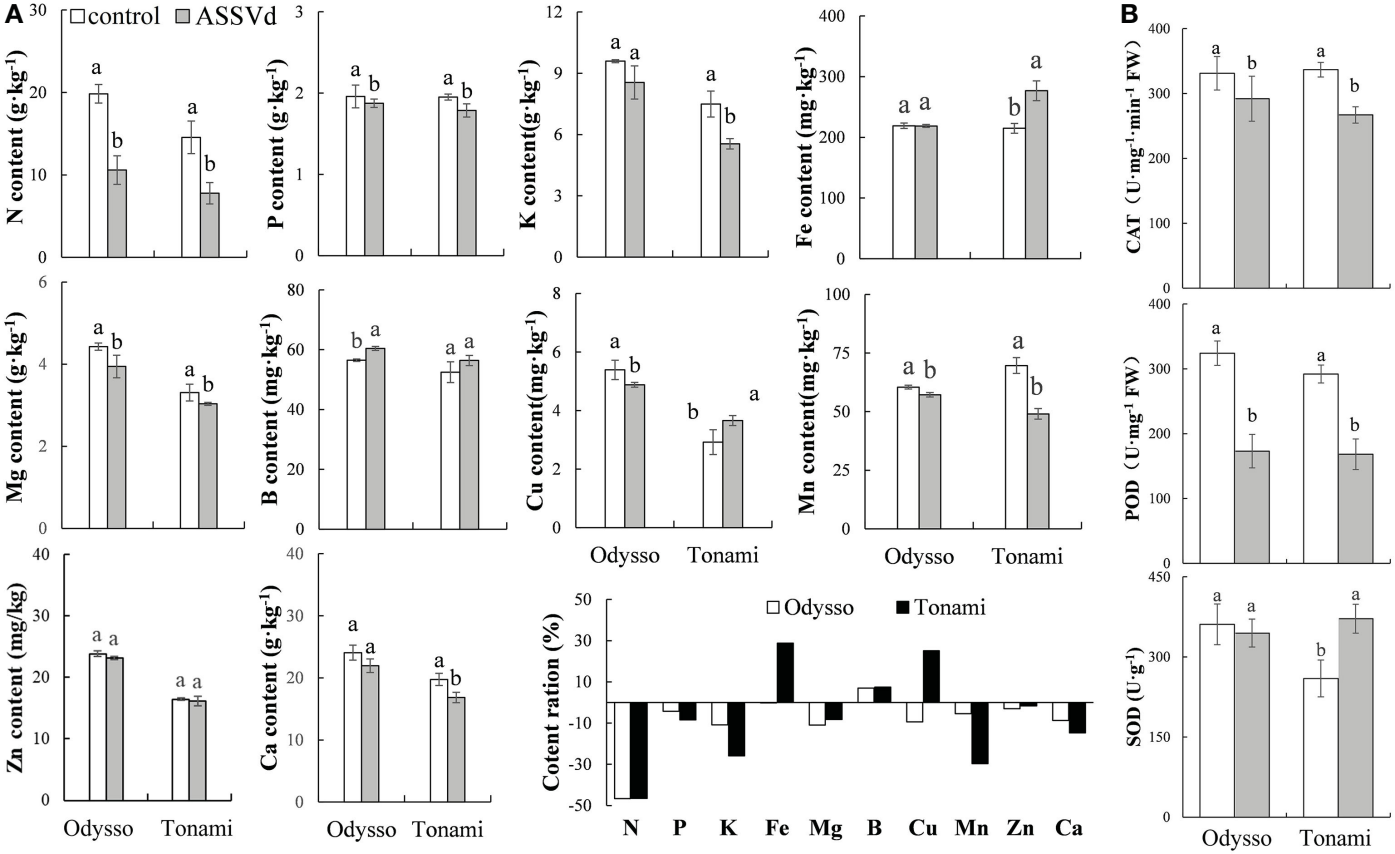
Effects of ASSVd on the nutrient element and antioxidant enzyme contents in leaves. **(A)** N, P, K, Fe, Mg, B, Cu, Mn, Zn, and Ca contents and ratios in the OY leaves of ‘Odysso’ and ‘Tonami’. **(B)** CAT, POD, and SOD contents in OY leaves. Data are presented as the mean ± SD (n=3). Lowercase letters indicate significant differences as determined by Student’s *t*-test (*P* < 0.05).

Nutrient elements are essential for plant growth and development as well as the composition of plant tissues and organs. Compared with the control saplings, the N, Mg, Cu, and Mn contents in the viroid-infected ‘Odysso’ saplings decreased significantly by 46.63%, 10.98%, 9.43%, and 5.41%, respectively, while the B content increased significantly by 6.96% (**Figure 4A**). In the ‘Tonami’ leaves infected with ASSVd, the N, P, K, Mg, Mn, and Ca contents decreased by 46.58%, 8.47%, 25.89%, 29.65%, 8.29%, and 14.75%, respectively, whereas the Fe content increased by 30.18% (**Figure 4A**). The rank-order of the decrease in the nutrient element contents was N > Mg > K > Cu > Ca > Mn > P > Zn > Fe > B for viroid-infected ‘Odysso’ and N > Mn > K > Ca > P > Mg > Zn > B > Cu > Fe for viroid-infected ‘Tonami’ (**Figure 4A**).

Compared with the controls, the leaf CAT and POD contents were lower for the viroid-infected saplings (**Figure 4B**), suggestive of a decrease in oxidative stress resistance. However, the SOD content was lower in the control ‘Tonami’ saplings than in the viroid-infected ‘Tonami’ samples (**Figure 4B**).

### Effects of ASSVd on the leaf metabolome

**Figure 5:**
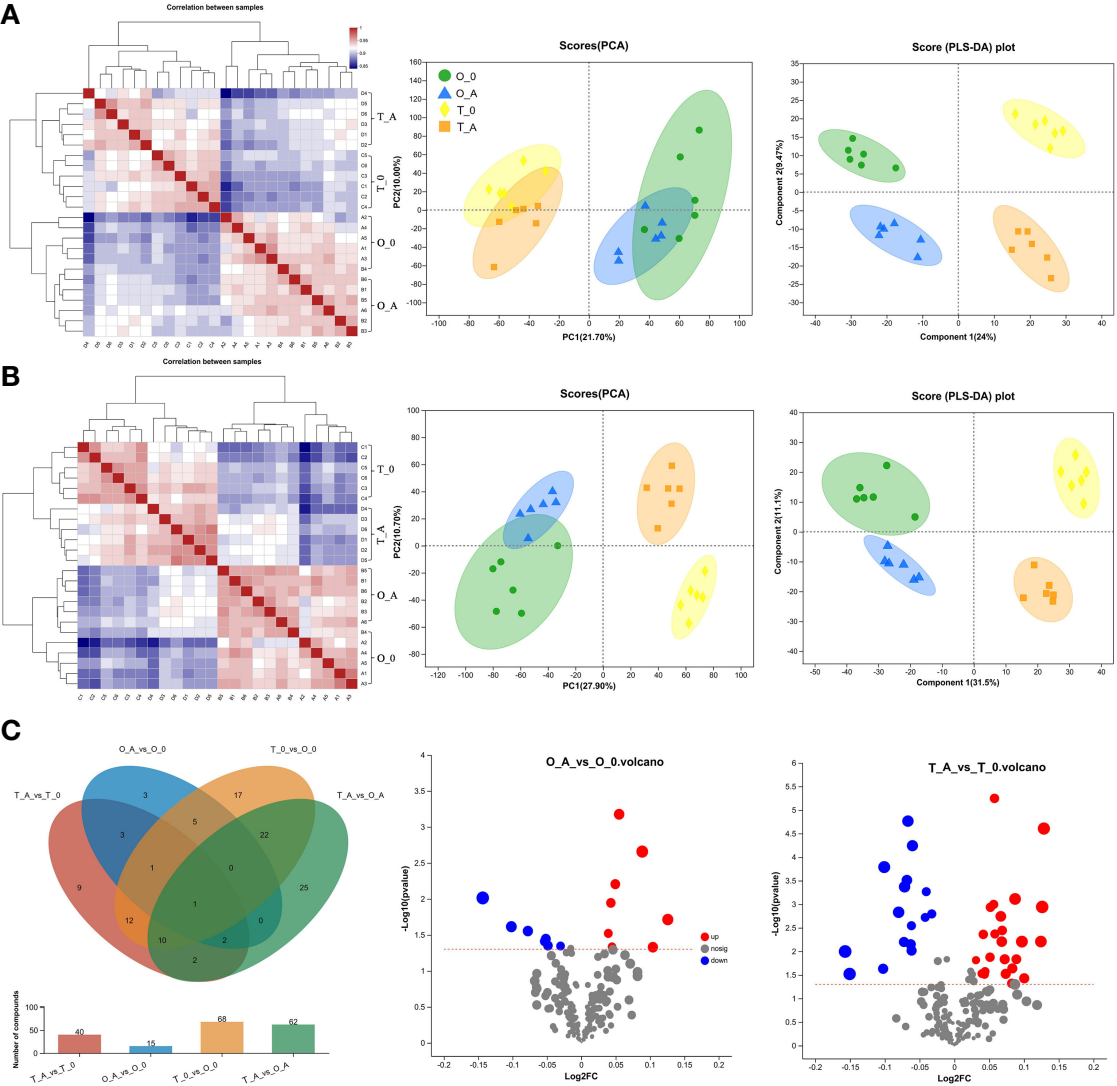
Heatmap, PCA and PLS-DA scores, and differentially abundant metabolites in leaves. Analysis of the positive **(A)** and negative **(B)** metabolites. In the heatmap, each lattice represents the correlation between two samples. The different colors represent the correlation coefficient between samples, whereas the length of the cluster branch represents the relative distance between samples. Each dot in the PCA and PLS-DA score maps represents the leaf metabolomic profile of a single sample. **(C)** In the Venn diagram, different colors represent different comparisons. Overlapping and non-overlapping numbers respectively represent the number of shared and unique metabolites. The bar graphs present the number of metabolites in the corresponding comparisons. In the volcano plot, the abscissa presents the fold-change in the metabolite abundance between two samples, whereas the ordinate presents the significance of the difference. Red, blue, and gray dots represent the up-regulated, down-regulated, and unchanged metabolites, respectively.

To characterize the effects, the content and species of metabolites in the leaves from ‘Odysso’ and ‘Tonami’ OY saplings were analyzed. Of the 12,751-pos and 12,465-neg peaks detected during the metabolomics analysis of the viroid-infected and control saplings, 136-pos and 72-neg metabolites were identified (**Table S4**). According to the correlation heatmaps based on positive and negative ions, the correlation coefficients between biological replicates ranged from 0.91 to 0.96 and 0.89 (two replicates of the control ‘Odysso’) to 0.96 for the same samples (**Figure 5A, B**). On the basis of the PCA and PLS-DA data, the confidence level of six biological replicates for each sample exceeded 95%. Additionally, different samples could be distinguished (**Figure 5A, B**). These results indicated the high-quality metabolomics data were suitable for the subsequent analysis.

**Figure 6:**
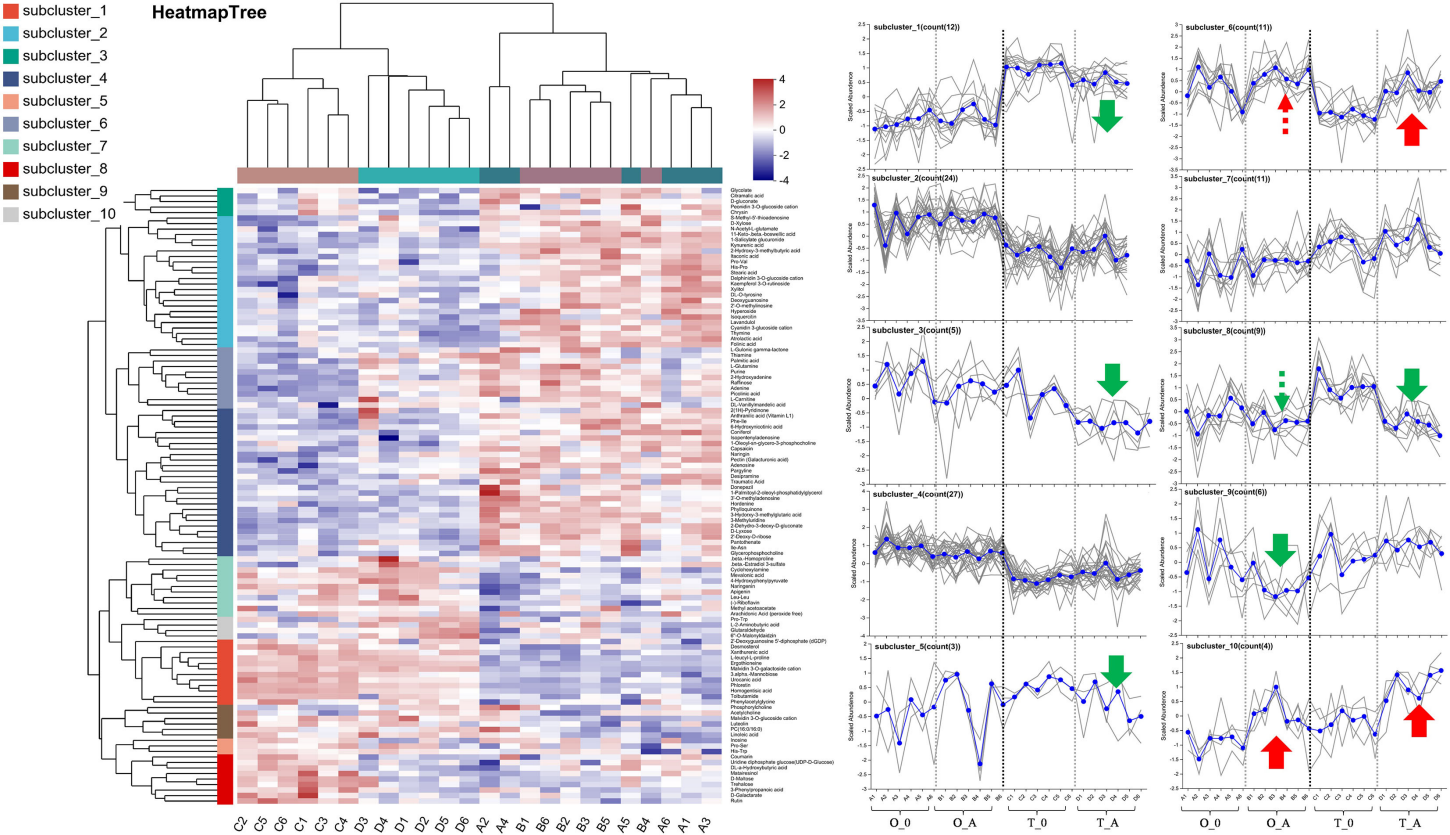
Cluster analysis of the leaf metabolites affected by the ASSVd infection. Each column represents a sample (code), whereas each row represents a metabolite (name). The color indicates the relative metabolite abundance. The dendrograms of the metabolite subclusters and sample clusters are presented on the left and at the top, respectively. The metabolite abundance in each subcluster was analyzed using vertical and horizontal coordinates representing the samples and the scaled abundance of metabolites, respectively. Green and red arrows indicate up-regulated and down-regulated metabolites, respectively. The solid and dashed lines indicate significant changes and certain trends, respectively.

A Venn diagram revealed 15 and 40 differentially abundant metabolites between the viroid-infected ‘Odysso’ and ‘Tonami’ samples and the corresponding controls, respectively (**Figure 5C**, **Table S5**). More differentially abundant metabolites were detected by the comparisons between the two viroid-infected and two control saplings. Furthermore, eight and seven up-regulated and down-regulated metabolites, respectively, were identified in ‘Odysso’, whereas 24 and 16 up-regulated and down-regulated metabolites, respectively, were identified in ‘Tonami’ (**Figure 5C**). The metabolite cluster analysis divided the metabolites into 10 subclusters. The metabolites in subclusters 1, 2, 4, and 7 clearly differed among samples (**Figure 6**). More specifically, the abundance of the metabolites in subclusters 10 and 6 increased in ‘Odysso’ and ‘Tonami’ in response to the ASSVd infection, whereas the opposite trend was observed for the subcluster 8 metabolites (**Figure 6**). These findings confirmed the metabolomes of the two apple varieties vary, but the ASSVd infection-induced changes in the contents of some metabolites were consistent between ‘Odysso’ and ‘Tonami’.

**Figure 7:**
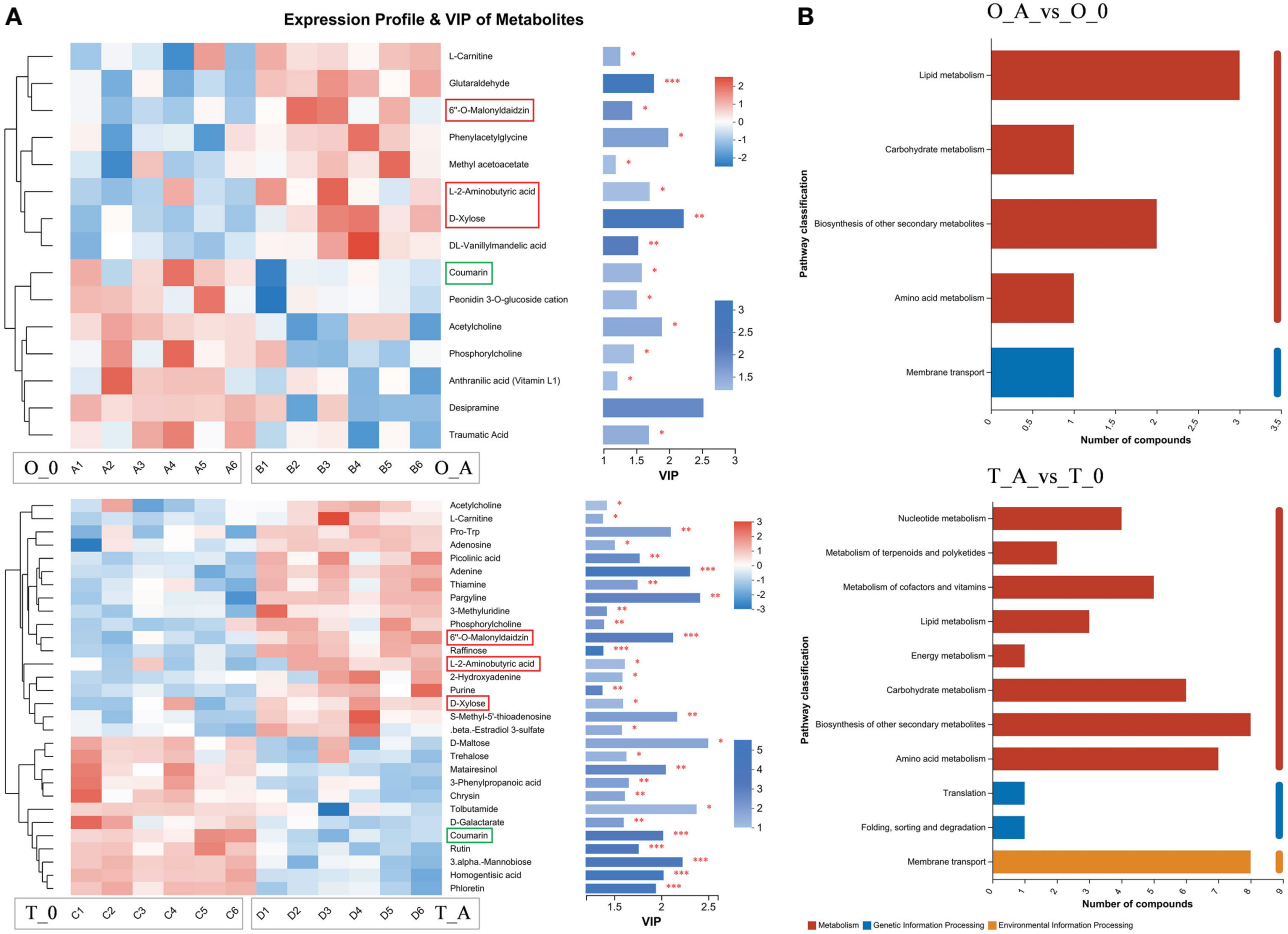
Heatmap and KEGG analysis of differentially abundant metabolites. **(A)** Left: metabolite cluster dendrogram, with each column representing one sample and each row representing one metabolite; the color indicates the relative metabolite abundance. Right: bar length indicates the contribution of the metabolite to the difference between samples. The blue bar indicates the significance of the differences in the metabolites between samples (*P < 0.05, **P < 0.01, and ***P < 0.001). **(B)** The ordinate presents the KEGG metabolic pathways, whereas the abscissa presents the number of metabolites. Red, blue, and yellow bars indicate the pathways belong to the Metabolism, Genetic Information Processing, and Environmental Information Processing categories.

Compared with the controls, the L-2-aminobutyric acid, 6″-O-malonyldaidzin, and D-xylose contents increased in the viroid-infected ‘Odysso’ and ‘Tonami’ samples, which was in contrast to the decrease in the coumarin content (**Figure 7A**). The VIP values of these four metabolites were significantly greater than 1, indicative of significant differences between the viroid-infected and control saplings (**Figure 7A**). The KEGG enrichment analysis revealed the differentially abundant metabolites between the viroid-infected and control saplings were significantly associated with lipid metabolism, carbohydrate metabolism, the biosynthesis of other secondary metabolites, amino acid metabolism, and membrane transport (**Figure 7B**).

## Discussion

### ASSVd affects apple sapling growth, photosynthetic activity, and nutrient element contents

Symptoms of an ASSVd infection are not appeared on the apple leaves and stems (Kim et al., 2010; Wu et al., 2015; Di Serio et al., 2018). However, leaf curling and leaf discoloration (e.g., faded and mottled) are included among the symptoms on litchi plants infected with ASSVd; however, these symptoms may not develop in some varieties (Jiang et al., 2017). An infection by ASSVd may weaken persimmon trees (Ito et al., 2013). In the current study, prior to detecting and quantifying the ASSVd content, it was difficult to determine the cause of the phenotypic changes in the examined saplings. In the viroid-infected ‘Odysso’ and ‘Tonami’ saplings, the decrease in Pn, Gs, and Tr may have been primarily due to the decrease in the chlorophyll content in the ‘Odysso’ leaves and the decline in photosynthetic efficiency in the ‘Tonami’ leaves (no change in the chlorophyll content) (Wang et al., 2018). These observations indicate that ASSVd infections can decrease the chlorophyll content or the photosynthetic efficiency in apple leaves, which may explain the decreased tree height, stem diameter, and total branch length detected for the viroid-infected ‘Odysso’ and ‘Tonami’ samples. In addition, Mg, Fe, and Cu are involved in the synthesis of chlorophyll (Gong et al., 2020). Compared with the ‘Odysso’ leaves, the Fe and Cu contents were significantly higher in the ‘Tonami’ leaves, which may help to explain the observed lack of change in the chlorophyll content.

The nutrient status of plants reportedly influences disease resistance (Williams and Salt, 2009). In the present study, the N, P, K, Mg, Mn, Zn, and Ca contents were significantly lower in the ASSVd-infected leaves than in the control leaves. Insufficient amounts of some of these nutrient elements may adversely affect the uptake of the other elements, thereby exacerbating the detrimental effects of ASSVd on plants (Gong et al., 2020). Several studies confirmed that these elements are involved in the biosynthesis of certain compounds, enzymatic reactions, and metabolic pathways (Liang et al., 2017; Gong et al., 2020). Accordingly, the ASSVd-induced changes in the contents of these elements may have contributed to the inhibited growth of the viroid-infected ‘Odysso’ and ‘Tonami’ saplings.

### ASSVd affects the leaf metabolomes of young apple saplings

Metabolomics-based analyses may identify important metabolites in specific physiological periods or conditions (Goodacre et al., 2004). Metabolic changes may be closely related to plant physiological performance. Recently reported changes in metabolites have helped to elucidate plant responses to various stresses (Moussaieff et al., 2013; Booth et al., 2015; Pidatala et al., 2016; Han et al., 2022). An infection by ASSVd may affect multiple tissues by altering nutrient accumulation and distribution throughout the plant. In apple saplings, the leaves are the main organs in which nutrients are synthesized, but they are also the primary tissues affected by ASSVd. In this study, 15 and 40 differentially abundant metabolites were identified in the ‘Odysso’ and ‘Tonami’ leaves, respectively, which implies that ASSVd has relatively limited effects on leaf metabolites. According to the KEGG analysis, most of the differentially abundant metabolites are associated with lipid, carbohydrate, and amino acid metabolism and the biosynthesis of other secondary metabolites. Thus, an ASSVd infection of ‘Odysso’ and ‘Tonami’ leaves significantly modulates specific metabolic pathways.

In the pathway mediating starch and sucrose metabolism, D-xylose is an intermediate during the synthesis of sucrose, which serves as a source of energy for various plant metabolic pathways and activities (Pan et al., 2016). We observed that the D-xylose content increased significantly in the ‘Odysso’ and ‘Tonami’ leaves, which reflected enhanced respiration and a decrease in photosynthetic productivity. A network analysis identified three metabolites (L-2-aminobutyric acid, 6″-O-malonyldaidzin, and coumarin) associated with signal transduction or stress resistance pathways. These changes in metabolite contents in ‘Odysso’ and ‘Tonami’ might be part of a specific response to an ASSVd infection. The differences in the phenotypes, photosynthetic activities, nutrient element contents, and metabolite contents between ‘Odysso’ and ‘Tonami’ suggest different apple varieties vary regarding their response to ASSVd. Compared with other apple varieties, the ASSVd-infected ‘Odysso’ saplings in this study had a lower ASSVd content, longer branches, fewer differentially abundant metabolites, and a greater anthocyanin content (i.e., 30% higher) (Pomiferous.com). However, determining the main common effects of ASSVd on apple trees may provide the theoretical basis for characterizing the mechanism mediating ASSVd infections and disease symptom development.

## Data availability statement

The original contributions presented in the study are included in the article/supplementary material. Further inquiries can be directed to the corresponding authors.

## Author contributions

GFL, JHL, HZ, YNW, MT, and JZS participated in the data analysis. JHL, LGJ, SWZ, JYL, JSS and JZS performed material sampling, field measurements and the laboratory data measurement. GFL, JHL, YNW, JSS, MT and JZS participated in the paper writing and manuscript amending. All authors contributed to the article and approved the submitted version.
