# Supplementary material for: ASSVd infection inhibits the vegetative growth of apple trees by affecting leaf metabolism
Source: Front Plant Sci. 2023 Feb 24;14:1137630. doi: 10.3389/fpls.2023.1137630 (PMC9998556; doi:10.3389/fpls.2023.1137630)
Supplement: Supplementary file 1 [file DataSheet_1.docx]

**Table S1 Sequence of PCR primers for ASSVd**

| Name | Sequence（5′to3′） | Products (bp) |
| --- | --- | --- |
| ASSVd-F | ACGAGGAGAAGAAGGGACTCAC | 106 |
| ASSVd-R | AACCCACAGCGGAACTGGT |  |
| ASSVd-f | CCGGATCCGGTAAACACCGTGCGGTCCC | 330 |
| ASSVd-r | CCGGATCCGGGAAACACCTATTGTGTTT |  |

**Table S2 Reproducibility assay of the qRT-PCR**

| cRNA Concentration (1.24 × ng·μl^-1^) | Reproducibility of intra-assay | | | |  | | Reproducibility of inter-assay | | |
| --- | --- | --- | --- | --- | --- | --- | --- | --- | --- |
|  | Mean Ct | SD | CV/% |  | | Mean Ct | | SD | CV/% |
| 10^11^ | 21.79 | 0.14 | 0.62 |  | | 27.34 | | 0.35 | 1.11 |
| 10^8^ | 32.25 | 0.24 | 0.75 |  | | 32.82 | | 0.42 | 1.27 |
| 10^5^ | 32.75 | 0.50 | 1.55 |  | | 34.64 | | 0.64 | 1.81 |

**Table S3 Effects of ASSVd on the shoot length in apple saplings**

| Samples | Short (cm) | Medium (cm) | Long (cm) | Super long (cm) | Height (cm) | Total length (cm) |
| --- | --- | --- | --- | --- | --- | --- |
| Od-control^NG^ | 40 | 70 | 80 | 120 | 136 | 446 |
| Od-ASSVd^NG^ | 4 | 60 | 117 | 0 | 134 | 315 |
| To-control^NG^ | 65 | 72 | 110 | 41 | 133 | 421 |
| To-ASSVd^NG^ | 45 | 50 | 25 | 76 | 101 | 297 |
| Od-control^OY^ | 5 | 177 | 474 | 2816 | 229 | 3503 |
| Od-ASSVd^OY^ | 5 | 482 | 537 | 1277 | 211 | 2512 |
| To-control^OY^ | 0 | 186 | 408 | 1471 | 214 | 2279 |
| To-ASSVd^OY^ | 5 | 154 | 240 | 1209 | 179 | 1878 |

**Table S4 Number of total ions and identified metabolites**

| Ion mode | All peaks | Identified metabolites |
| --- | --- | --- |
| positive | 12751 | 136 |
| negative | 12465 | 72 |

**Table S5 Differentially abundant metabolites in the ‘Odysso’ and ‘Tonami’ leaves**


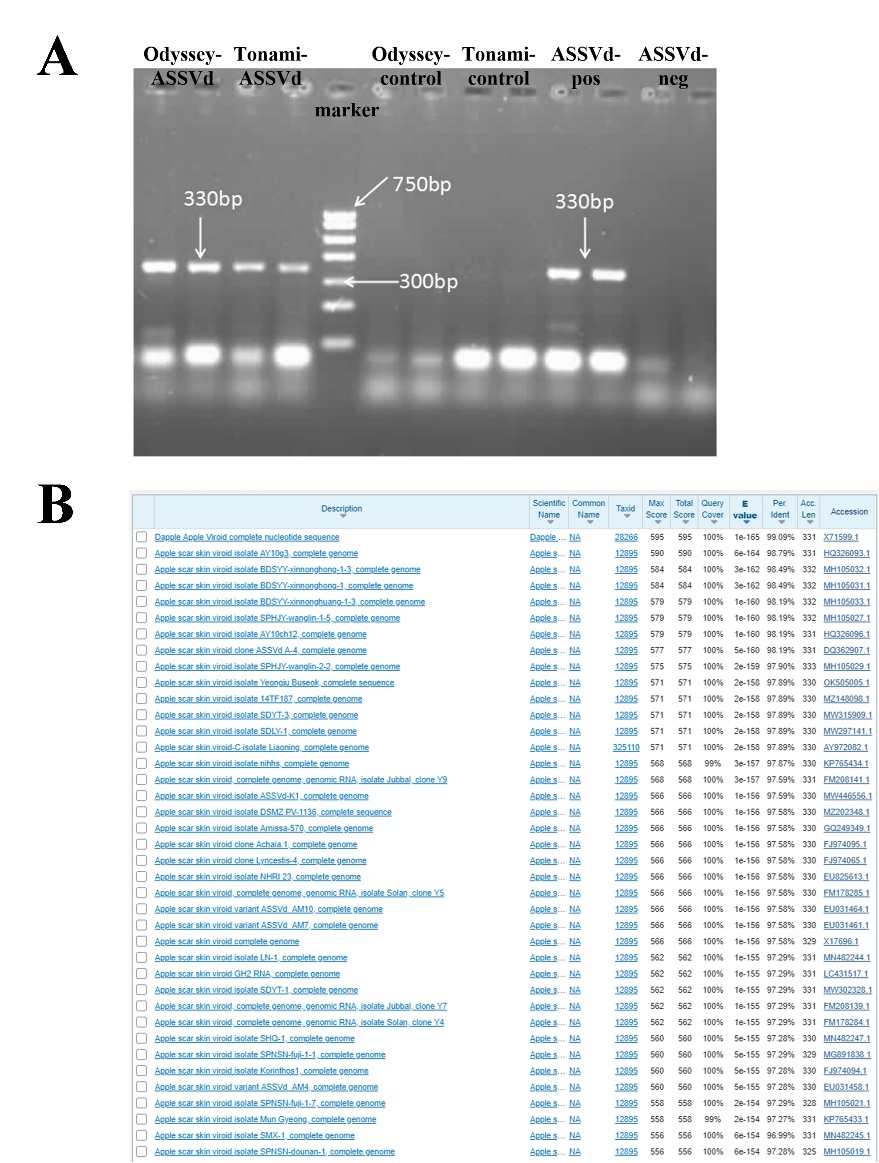


**Figure S1 PCR detection and alignment of ASSVd and other sequences.** (A) Gel electrophoresis of ASSVd amplified by PCR. (B) Alignment of the sequences similar (>97%) to the ASSVd sequence according to the Nucleotide BLAST search of the NCBI database.
